# Repeat Discectomy for the Management of Same-Level Recurrent Disc Herniation: A Study of 50 Patients

**DOI:** 10.7759/cureus.40469

**Published:** 2023-06-15

**Authors:** Gerald Musa, Serik K Makirov, Sergey V Susin, Gennady E Chmutin, Alexandre V Kim, Dmitri V Hovrin, Dimitri T.K Ndandja, Olzhas B Otarov, Hesham M Shaafal, Karina Familia Ramirez

**Affiliations:** 1 Neurological Surgery, Peoples' Friendship University of Russia (RUDN University) Named After Patrice Lumumba, Moscow, RUS; 2 Neurological Surgery, Livingstone Central Hospital, Livingstone, ZMB; 3 Trauma and Orthopaedics, Scientific and Technical Center, Family Clinic, Moscow, RUS; 4 General Surgery, Scientific and Technical Center, Family Clinic, Moscow, RUS; 5 Neurological Surgery, City Clinical Hospital Named After V.P Demikhov, Moscow, RUS; 6 Neurological Surgery, City Clinical Hospital Named After C.C Yudina, Moscow, RUS; 7 Orthopaedics and Trauma, Scientific and Technical Center, Family Clinic, Moscow, RUS

**Keywords:** discectomy, modic changes, spinal instability, degenerative disc disease, oswestry disability index (odi), recurrent lumbar disc herniation

## Abstract

Background

Same-level recurrent disc herniation remains a challenge in spine surgery. Although most surgeons agree on discectomy as the treatment of choice for primary lumbar disc herniation, the management of recurrent disc herniation remains ambiguous and largely depends on the operating surgeon. Many surgeons recommend repeat discectomy over fusion because it is cheaper and less invasive. In this study, we analyzed 50 patients who underwent a repeat discectomy.

Materials and methods

The patients in the study had previously been managed for lumbar disc herniation and then presented with either recurrent same-level herniation or symptoms attributed to the same level. The patients were then managed with a repeat discectomy without fusion. We analyzed the preoperative and postoperative Oswestry Disability Index (ODI), duration of surgery, blood loss, duration of hospitalization, and complications.

Results

Fifty patients were included: 27 females (54%), and 23 males (46%). They were followed up for an average of 2.81 years (range: 1-4). The mean duration of hospitalization was 4.06 ± 1.5 days (range: 2-8). The operative time was 104.60 minutes (range: 50-195), with an intraoperative blood loss of 85.40 mL (range: 50-150 mL). Durotomy occurred as a complication in eight (16%) patients. The recurrence rate was 26%, with 36% progressing to fusion. The change in preoperative ODI and postoperative ODI was 20.94 ± 7.24 (6-37), with a p-value of 0.04. There were no long-term complications recorded.

Conclusion

Repeat discectomy is a good management option for same-level recurrent disc herniation. The procedure is associated with low intraoperative blood loss and a short operating time, but there is a significant risk of durotomy. The risk of recurrence remains a concern due to the progression of degenerative changes, especially in the presence of Modic-2 changes. These advantages and disadvantages should be discussed with patients.

## Introduction

Degenerative disc disease and facet joint problems affecting the lower back are common in the aging population and are major causes of disability. Statistics indicate that approximately 40% of individuals over 40 years old and up to 80% of those over 80 years old suffer from this condition [1]. However, it should be noted that the onset of degeneration is not limited to middle-aged and elderly people [2]. Symptoms of this condition include mechanical back pain, radicular pain, claudication, reduced mobility, and a decreased quality of life [3].

A common manifestation of degenerative spine disease is a herniated disc, which is typically treated through discectomy. Several discectomy methods have been developed, including conventional open surgery, microdiscectomy, endoscopic discectomy, and others [4,5]. However, it is important to note that the recurrence of disc herniation following discectomy is between 10% and 30%, and progression to instability occurs in about 25% of cases [6-8]. Although discectomy is the standard treatment for a first-time herniated disc, the management of recurrent herniations is still a matter of debate. Some authors suggest repeat discectomy due to its minimally invasive nature, shorter hospital stay, and cost-effectiveness [6,9]. However, this approach still carries the risk of re-herniation and progression to instability, which can worsen the pain score [10-12]. Fusion techniques, on the other hand, eliminate the risk of same-level recurrence and segment instability [13-15]. The debate against fusion stems from the cost of the implants, a longer hospital stay, prolonged surgery time, and increased blood loss [4]. Despite potential disadvantages, some authors believe that fusion techniques are necessary for managing recurrent herniation [15,16]. This study analyzes the use of repeat discectomy in the management of same-level lumbar disc re-herniation.

## Materials and methods

This study is a retrospective case series conducted at a single center, including a total of 50 patients who experienced recurrent disc herniation and underwent repeat discectomy without fusion between the years 2018 and 2022. Recurrent intervertebral disc herniation was defined according to the criteria established by Yao et al. in 2016 [5]. The definition required that the patient had previously undergone a successful discectomy without fusion surgery. Additionally, the patient needed to be free of pain for at least one month after the initial surgery and exhibit recurring symptoms consistent with the affected level. Furthermore, confirmation of disc herniation recurrence at the same level as the previous discectomy surgery was required through magnetic resonance imaging (MRI) evaluation. Patients who experienced recurring pain or had disc herniation within one month of the surgery were excluded from the study, as this was considered surgical failure rather than recurrence. Patients with herniations at a different level than the one previously operated on were also excluded. The study collected the following information: demographic data (such as age and sex), radiological data (including the level of the lumbar spine and the presence of Modic changes on preoperative MRI), clinical data (presenting complaints, comorbidities, duration of follow-up, preoperative and postoperative pain levels, and Oswestry Disability Index), and surgical history (type of discectomy, number of repeat discectomies, intraoperative blood loss, operative time, complications, duration of hospitalization, and recurrence). The statistical analysis was performed using the IBM Statistical Package for the Social Sciences (IBM SPSS) version 26, Armonk, New York, United States, with significance values set at a p-value of less than 0.05. This threshold was chosen to determine the statistically significant findings in the data analysis.

## Results

There were a total of 50 patients included in this study. Of these participants, 27 (54%) were female, while 23 (46%) were male. The mean duration of follow-up was 2.81 years (1-4). The mean age was 50.94 years (33-72). The mean ages for males and females were 50.75 and 51.15 years, respectively (Table 1).

**Table 1:** Showing analysis of age in both the male and female patients. N: Number of patients; std: Standard.

Sex	Mean	N	Std. Deviation	Minimum	Maximum	Std. Error of Mean
Female	51.15	27	8.839	33	66	1.701
Male	50.70	23	10.306	34	72	2.149
Total	50.94	50	9.445	33	72	1.336

**Figure 1:**
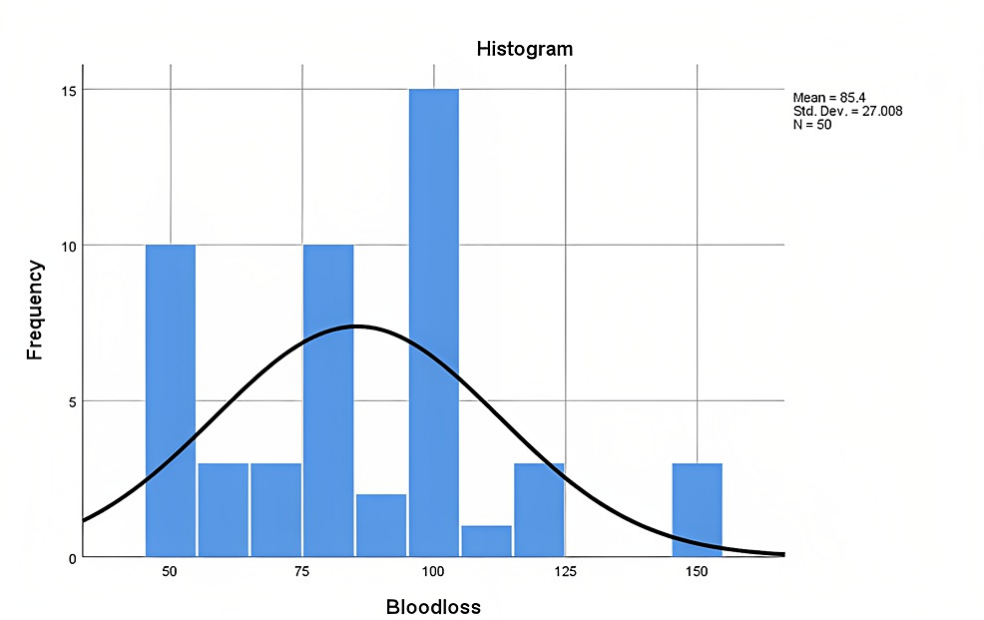
Showing the distribution of intraoperative blood loss. N: number of patients; Std. dev.: standard deviation.

**Figure 2:**
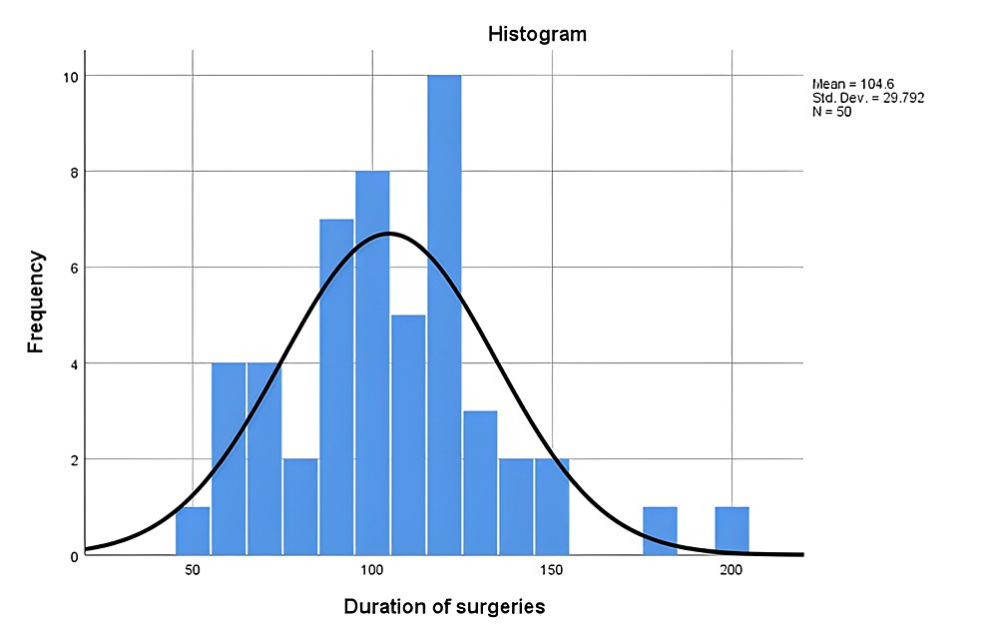
Showing the duration of surgery. N: number of patients; Std. Dev: standard deviation.

The type of discectomy the patient underwent prior to this study was recorded. The patients previously underwent microscopic discectomy (36%), open discectomy (50%), and endoscopic discectomy (14%). Forty percent of the patients had one previous discectomy, while 60% had multiple discectomies. The highest number of discectomies was five. Upon admission to our institution, the patients underwent either an open discectomy (58%; n = 29) or a microdiscectomy (42%; n = 21). The mean intraoperative blood loss was 85.40 ml (50-150 ml) (Figure 1), while the mean operative time was 104.60 minutes (50-195) (Figure 2).

**Figure 3:**
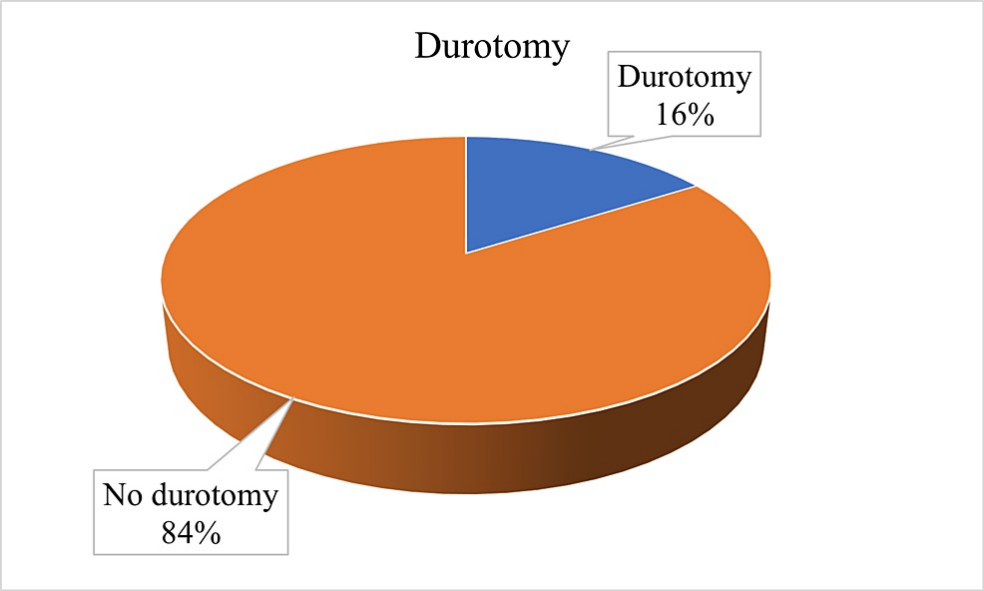
Showing the percentage of incidental durotomy.

Surgical time was defined as the time from the skin incision to the placement of the last suture on the skin closure. The only intraoperative complication was incidental durotomy, which was present in 16% (n = 8) (Figure 3).

**Figure 4:**
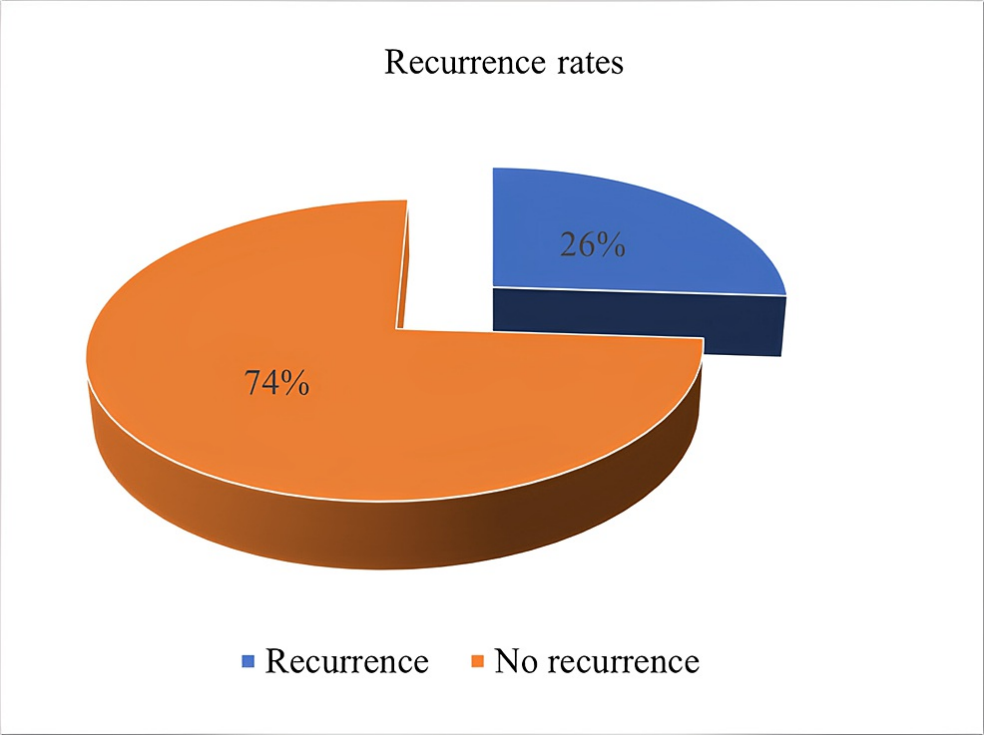
Showing the percentage of patients with recurrence.

Analysis of the preoperative MRI showed Modic-2 changes in 78% (n = 39). Of the 50 patients, 26% (n = 13) had a recurrence following the repeat discectomy (Figure 4).

**Figure 5:**
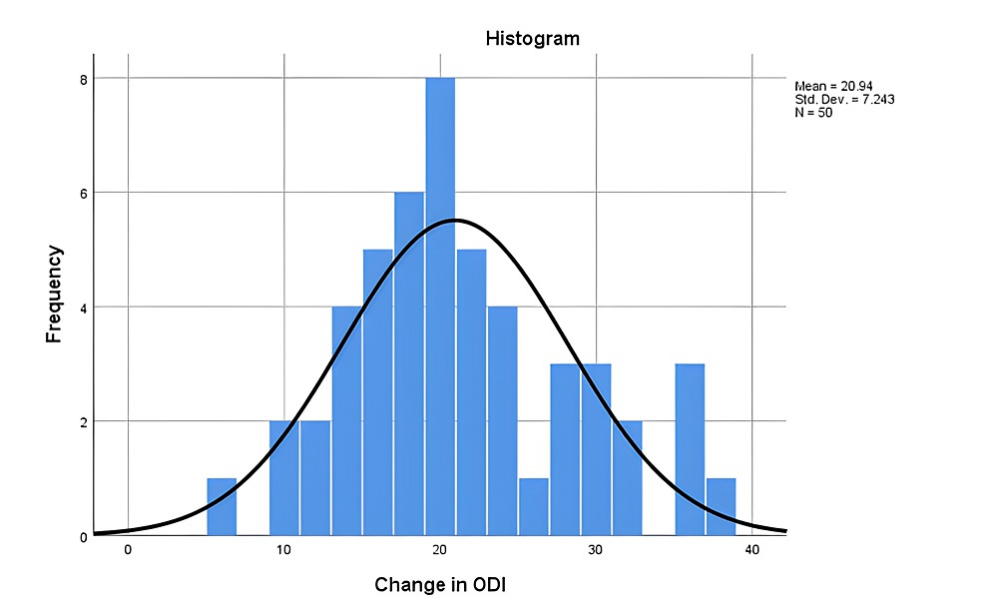
Showing the change in preoperative and postoperative Oswestry Disability Index (ODI). N: number of patients; Std. Dev: standard deviation; ODI Oswestry Disability Index.

Of note is that 83% of these patients had Modic-2 changes on preoperative MRI. Progression to fusion following repeat discectomy was eventually performed in 36% of patients during the follow-up period. The mean preoperative ODI was 30.52 (16-41), while the postoperative mean ODI was 9.58 (0-26). The change in preoperative ODI and postoperative ODI was 20.94 ± 7.24 (6-37) (Figure 5).

The difference was significant (p-value = 0.04). The duration of hospitalization was 4.06±1.5 days (2-8 days).

## Discussion

Recurrent lumbar disc herniation (RLDH) is a challenge in spinal surgery. There is still no standardized international protocol for the management of these patients. With the development of various equipment and technology in spinal surgery, the options for approaches to managing lumbar intervertebral disc herniations have increased [5]. These include the standard open discectomy, microscopic discectomy, and several variations of endoscopic discectomy.

Any repeat operation comes with additional risks compared to first-time surgeries. The presence of adhesions and distortion of anatomy makes identification and dissection of the structures a challenge [3]. In RLDH surgery, these adhesions increase the risk of injury to the dura and spinal nerves. The risk of durotomy ranges from 2% [17] to 16% [18,19]. This is similar to the findings in this retrospective review (16%). To avoid these adhesions, some authors have suggested a more lateral approach with a total facetectomy [20] or the use of antifibrin chemicals [21] during the first surgery. However, facetectomy may lead to instability, and the benefit of antifibrin material is not proven. One of the most challenging complications of repeat discectomy is the risk of re-herniation. There is a lot of variability in the literature on the rates of recurrence depending on the duration of follow-up, ranging from 2.4% [22] to 56% [5]. In this study, the recurrence was 26%. Disc herniation is a late feature of degenerative spine disease and a sign of spinal segment instability. This means that, in the absence of stabilization, the pathological micromovements at this segment will predispose to same-level and adjacent-level recurrent herniation, deformity, and pain [23]. As a result, progression to fusion is reported at up to 83% [24]. In this study, 36% of the patients progressed to fusion during the four-year follow-up period. Despite this, repeat discectomy has good postoperative pain control with a significant improvement in ODI (p = 0.04).

Repeat surgeries usually require longer operative times due to the difficulties in tissue dissection and the risks of intraoperative complications and hemorrhage [25]. This study showed a relatively short operation time of 104.60 ± 45.44 min (50-195), similar to what is reported in the literature [3,18,26]. However, it should be noted that the duration depends on multiple factors, including the expertise of the operating surgeon, whether the surgeon performs a sequestrectomy or discectomy, whether the recurrence is on the same side as the previous surgery or on the opposite side, etc. [27]. Intraoperative blood loss can be a concern in repeat surgery. In this study, the mean blood loss was 85.40 ml (50-150 ml), similar to what is reported in the literature, i.e., minimal or unmeasurable to 20 ml and 70-150 ml for endoscopic and microdiscectomy, respectively. A significant finding in this study was the relationship between recurrence and Modic changes. The presence of Modic-2 changes has been associated with lumbar motion segment instability [28-30]. This study showed that 83% of patients with recurrences had Modic-2 changes. On the other hand, only 40% of patients who did not re-herniate had Modic changes on preoperative MRI. This is very important, as other management techniques like fusion should be considered in these patients instead of discectomy due to the increased risk of recurrence. Similar to what has been reported in the literature, the length of stay was 4.06 ± 1.5 days. The patients are usually kept in admission for postoperative pain control and observation. Some of the advantages of repeat discectomy over fusion surgeries for lumbar disc herniation include shorter hospitalization, faster recovery, and a cheaper operation with no need for implants. Fusion surgery is also associated with a higher risk of adjacent segment disease, especially when lumbar lordosis is not corrected. This analysis is beyond the scope of this study.

This study has some limitations. The number of patients is small; a larger multicenter study would provide more information. The absence of a comparative group is another weakness of the study. This study analyzes the single-center experience of repeat discectomy. In the future, a multicenter study comparing various management options will be more informative in creating a management protocol.

## Conclusions

Repeat discectomy is a good management option for same-level recurrent disc herniation. The procedure is associated with low intraoperative blood loss and a short operating time. However, there is a significant risk of durotomy. The risk of recurrence remains a concern due to the progression of degenerative changes, especially in the presence of Modic-2 changes. These advantages and disadvantages should be discussed with the patients.
